# Chrysin-Loaded Chitosan Nanoparticles Potentiates Antibiofilm Activity against *Staphylococcus aureus*

**DOI:** 10.3390/pathogens9020115

**Published:** 2020-02-12

**Authors:** Busi Siddhardha, Uday Pandey, K. Kaviyarasu, Rajasekharreddy Pala, Asad Syed, Ali H. Bahkali, Abdallah M. Elgorban

**Affiliations:** 1 Department of Microbiology, School of Life Sciences, Pondicherry University, Puducherry 605014, India; uday.pandey@niser.ac.in; 2 Nanosciences African Network (NANOAFNET), Materials Research Group (MRG), iThemba LABS-National Research Foundation (NRF), Old Faure Road, P.O. Box 722, Somerset West 7129, South Africa; kasinathankaviyarasu@gmail.com; 3 Department of Biomedical & Pharmaceutical Sciences, Chapman University, School of Pharmacy, Irvine, CA 92618-1908, USA; rrpala@chapman.edu; 4 Department of Botany and Microbiology, College of Science, King Saud University, P.O. 2455, Riyadh 11451, Saudi Arabia; abahkali@ksu.edu.sa (A.H.B.); aelgorban@ksu.edu.sa (A.M.E.)

**Keywords:** biofilm, chitosan, chrysin, nanoparticles, *Staphylococcus aureus*

## Abstract

The application of nanotechnology in medicine is gaining popularity due to its ability to increase the bioavailability and biosorption of numerous drugs. Chrysin, a flavone constituent of *Orocylumineicum vent* is well-reported for its biological properties. However, its therapeutic potential has not been fully exploited due to its poor solubility and bioavailability. In the present study, chrysin was encapsulated into chitosan nanoparticles using TPP as a linker. The nanoparticles were characterized and investigated for their anti-biofilm activity against *Staphylococcus aureus*. At sub-Minimum Inhibitory Concentration, the nanoparticles exhibited enhanced anti-biofilm efficacy against *S. aureus* as compared to its bulk counterparts, chrysin and chitosan. The decrease in the cell surface hydrophobicity and exopolysaccharide production indicated the inhibitory effect of the nanoparticles on the initial stages of biofilm development. The growth curve analysis revealed that at a sub-MIC, the nanoparticles did not exert a bactericidal effect against *S. aureus*. The findings indicated the anti-biofilm activity of the chrysin-loaded chitosan nanoparticles and their potential application in combating infections associated with *S. aureus*.

## 1. Introduction

Biofilms—aggregations of densely packed microbial cells embedded inside exopolysaccharide (EPS) matrix—are a major challenge in public health management. The EPS matrix provides a protective barrier to the biofilm, making them recalcitrant to antimicrobial agents and host defenses [1]. *Staphylococcus aureus* is thought to be one of the major causes of nosocomial infections, globally. While the planktonic counterpart is limited to bacteremia and skin abscesses, more chronic infections such as cystic fibrosis, osteomyelitis, and endocarditis are associated with its biofilm mode of growth [2,3]. To eradicate this problem, many strategies were proposed, including (i) the inhibition of primary bacterial adhesion or attachment to the living or non-living surfaces; (ii) the disruption of biofilm architecture during maturation processes; and (iii) the inhibition of cell to cell communication—i.e., quorum sensing [4,5]. Chrysin (5, 7-dihydroxyflavone), a flavone constituent of the *Orocylumineicum vent* has already been documented for its anticancer, antioxidant and antibacterial properties [6,7]. In spite of its many biological activities, its low water solubility, poor biosorption in the intestinal lumen, low bioavailability and rapid metabolism in the body limits its therapeutic applications [8]. In this regard, a reduction in particle size may serve as a potential means for enhancing the solubility and dissolution of chrysin [9].

Nanocarriers have the ability to inhibit bacterial growth and biofilm formation, and are increasingly being used as an attractive tool to combat chronic infections [10]. They aid in increasing the efficacy of the drug by acting as a protective barrier against enzymatic hydrolysis, increase the biosorption efficacy of the drug in the intestinal lumen, increase solubility and also cause sustainable release [11,12]. In recent years, chitosan is widely being used as a nano-carrier due to its non-toxicity, biocompatibility, immunostimulating and mucoadhesive properties [13]. Chitosan is a cationic heteropolysaccharide composed of the β-(1,4) linked repeating unit of glucosamine (GlcN) and N-acetylglucosamine (GlcNAc), extracted by the partial alkaline N-deacetylation of chitin found in the exoskeleton of crustaceans [14,15]. The antimicrobial and anti-biofilm potential of chitosan and its nano-derivatives were reported against various microorganisms such as *Listeria monocytogenes*, *Bacillus cereus*, *Enterococcus faecalis,* etc. [1,16]. Chrysin-encapsulated chitosan nanoparticles (CCNPs), synthesized using the ionic gelation method, were characterized and evaluated for their anti-biofilm activity against *S. aureus*.

## 2. Materials and Methods

### 2.1. Materials

Chitosan (75–85% deacetylated), sodium tripolyphosphate (TPP) and Chrysin were procured from Sigma-Aldrich. The test stain, *S. aureus* (MCC 2408) was purchased from Microbial Culture Collection (MCC), Pune, India.

### 2.2. Synthesis of Chrysin-Loaded Chitosan Nanoparticles

Medium molecular weight chitosan (0.2%, *w*/*v*) was mixed with an aqueous solution of acetic acid (0.1%, *v*/*v*) and incubated over-night at 60 °C with continuous agitation. A stock of 5 mg/mL of chrysin dissolved in DMSO was used to prepare the nanoparticle formulation. An aliquot of chrysin prepared in DMSO was added to the chitosan solution (pH 4.8). Subsequently, 40 mL of TPP solution (0.2%, *w*/*v*) was dispensed dropwise into the chitosan–chrysin solution and kept under continuous agitation at 1000× *g* for 30 min. The ratio of the chitosan-TPP was maintained at 5:1 [13] with the final chrysin concentration of 50 µg/mL. The nanoparticles formed were concentrated by centrifuging the suspension for 20 min at 12,000× *g*, washed with MilliQ water to remove the unbound chrysin and dried at room temperature for further studies [17].

### 2.3. Physical Characterization of Nanoparticles

The nanoparticles (NPs) were subjected to dynamic light scattering (DLS) to determine the mean hydrodynamic diameter (MHD) and polydispersity index (PDI). The FTIR spectrum was recorded in the range of 4000–500 cm^−1^. A transmission electron microscope (TEM) was used to determine the morphology and size of the CCNPs [18].

### 2.4. Determination of the Loading Efficiency and Drug Release of Chrysin-Loaded Chitosan NPs

The amount of chrysin loaded in the nanoparticles was determined using a UV-Vis spectrophotometer. After the collection of NPs from the reaction mixture, the absorbance of the supernatant was recorded at 348 nm and the concentration of unbound chrysin was estimated based on the standard curve of chrysin [13,19]. The CCNPs were dislodged in a medium constituting PBS and DMSO (co-solvent, 1%, *v*/*v*) and incubated with gentle agitation (100× *g*) at 37 °C. Two milliliters of the sample was retrieved at regular intervals, centrifuged at 10,000× *g* and the absorbance of the supernatant was recorded at 348 nm. The cumulative chrysin released in the medium was determined at the every 2 h interval, with reference to the standard curve of chrysin [20]. Chrysin release (%) = (Chrysin released in the supernatant/loaded chrysin concentration) × 100.

### 2.5. Determination Sub-Minimum Inhibitory Concentration (Sub-MIC) of Chrysin-Loaded Chitosan NPs

The minimum inhibitory concentration (MIC) of CCNPs was determined using macro-broth dilution assay (Clinical and Laboratory Standards Institute Guidelines, 2006). Two-fold dilutions of the NPs were prepared in Muller-Hinton broth to achieve a final concentration ranging from 8 µg/mL to 1024 µg/mL. An overnight culture of *S. aureus* (100 µL) was added in each NPs suspension and incubated at 37 °C for 24 h. The test tubes were observed for visible signs of growth and the spectrophotometric readings were recorded at 600 nm [12].

### 2.6. In Vitro Anti-Biofilm Assays of Chrysin-Loaded Chitosan NPs

The effect of sub-MIC of CCNPs of the on the biofilm formation of *S. aureus* was evaluated relative to chitosan NPs (CNPs) and Chrysin.

#### 2.6.1. Microtiter Plate (MTP) Assay for Biofilm Disruption and Inhibition

MTP assay for biofilm disruption and inhibition was performed according to Mu et al. [1]. An over-night culture of *S. aureus* (100 µL) was transferred into the wells of 96-well flat-bottomed polystyrene plates. After incubation at 37 °C for 24 h, the wells were washed with 100 µL of 0.9% (*w*/*v*) NaCl to remove the unadhered cells. The biofilm formed was further incubated after adding 90 µL tryptone soy broth (TSB) supplemented with the sub-MIC concentration of CCNPs for another 24 h. The biofilm attached at the bottom of each well was fixed with 100 µL of absolute methanol for 15 min and subsequently treated with 100 µL of crystal violet (0.2% *w*/*v*). Control samples were maintained with *S. aureus* culture alone. Cultures of *S. aureus* treated with DMSO serve as a control. The dye attached to the biofilm was further solubilized in 150 µL of glacial acetic acid and the optical density was recorded at 595 nm. *S. aureus* culture (90 µL) grown in TSB was seeded into individual wells of microtiter plates in the presence of sub-MIC of CCNPs and incubated at 37 °C for 24 h. The planktonic cells were discarded and the MTP was stained with crystal violet (0.2% *w*/*v*). The inhibition of biofilm formation was determined by solubilizing the CV attached to the biofilm and measuring the optical density at 595 nm. Biofilm inhibition/disruption was quantified using the following formula:

% Biofilm inhibition/disruption= ([OD_595_ of control − OD_595_ of test] = OD_595_ of control) * 100

#### 2.6.2. Microscopic Examination of Biofilm

Reduction in the biofilms of *S. aureus* was observed using confocal laser scanning microscopy (CLSM). *S. aureus* biofilms was allowed to grow on glass coverslips (18 × 18 mm) placed in 12 well polystyrene plates containing TSB supplemented with CCNPs (sub-MIC) and incubated overnight at 37 °C. The glass coverslip was washed with sterile distilled water, stained and processed accordingly [21]. The biofilm formed was fixed using methanol and treated with crystal violet (0.2% *w*/*v*). The coverslip was subsequently washed, air dried and observed using a light microscope (40×). The biofilm formed on the coverslip was washed 0.01 M phosphate buffer saline (PBS), stained using acridine orange (0.2% *w*/*v*) for 1 min and observed using Confocal laser microscope at 20×. The 3D image was recorded and Z stacks were prepared to determine the effect of the CCNPs on the thickness of the biofilm [22].

#### 2.6.3. Exopolysaccharide (EPS) Quantification and Microbial Adhesion to Hydrocarbon (MATH) Assay

Production of EPS by *S. aureus* was quantified in presence and absence of CCNPs by total carbohydrate quantification method. *S. aureus* was grown in presence and absences of CCNPs were harvested by centrifugation (10,000× *g* for 2 min). The cell pellet was washed and suspended in 200 µL of sterile PBS to which an equal volume of 5% (*v*/*v*) phenol and 5× volume of concentrated sulfuric acid containing 0.2% (*w*/*v*) hydrazine sulphate was added. The tubes were incubated in dark for 1 h followed by centrifugation at 10,000× *g* for 10 min. The supernatant was aspirated and the optical density was measured at 490 nm [21]. A reduction in EPS production was quantified using the following formula:

% EPS quantification=([OD490 of control−OD490 of test]÷OD490 of control)×100

The effect of CCNPs on the cell surface hydrophobicity of *S. aureus* was evaluated using MATH assay. The optical density of the treated CCNPs and the untreated cell suspension was recorded at 600 nm, after 24 h of incubation. The bacterial suspension was mixed with toluene (1 mL) and vortexed for 2 min. The optical density of the aqueous phase was measured at 600 nm. In both the assays, control samples were maintained with *S. aureus* culture only. Cultures of *S. aureus* treated with DMSO served as a negative control [21]. The percentage of inhibition in hydrophobicity is measured as follows;

$$\mathrm{Hydrobhobicity}\left(\%\right)=1-\frac{\mathrm{OD}600\;\mathrm{after}\;\mathrm{vortexing}}{\mathrm{OD}600\;\mathrm{before}\;\mathrm{vortexing}}\times 100$$

#### 2.6.4. Growth Curve Analysis 

An overnight culture of *S. aureus* was diluted with LB medium until the optical density of the cell suspension reaches 0.05 at 600 nm. The suspension was then supplemented with chrysin and NPs separately and incubated overnight at 37 °C at 100× *g*. The cell suspension (1 mL) was withdrawn and the optical density was measured at 600 nm, at every 2 h interval [23]. 

### 2.7. Statistical Analysis

All the assays were repeated thrice, and the data are presented as mean ± standard error. Significance among treatments were investigated using one-way ANOVA and represented with a statistical significance of *p* ≤ 0.05. Significance in treatments of chrysin, chitosan and CCNPs are represented with asterisk sign. Non-significant groups are represented by NS.

## 3. Results

### 3.1. Synthesis and Characterization of Chrysin-Loaded Chitosan NPs

**Figure 1:**
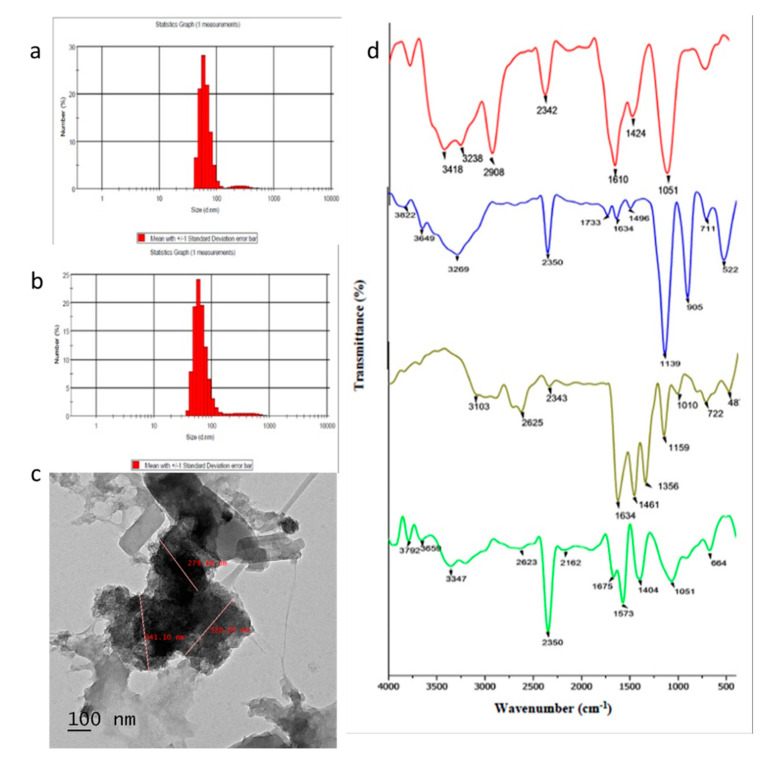
Characterization of NPs: Size distribution of (**a**) chitosan nanoparticles (CNPs) and (**b**) chrysin-encapsulated chitosan nanoparticles (CCNPs); (**c**) TEM micrograph showing polydispersed CCNPs with size range of 130–341 nm; (**d**) FTIR spectra of chitosan (red), TPP (blue), chrysin (grey) and CCNPs (green).

The CCNPs were synthesized using the ionotropic gelation method, using TPP molecules as a linker. The ratio of chitosan and TPP used is one of the factors that influence the aggregation of nanoparticles. Chitosan/TPP in the ratio of 5:1 was found to be the best formulation for the synthesis of CCNPs. The mean hydrodynamic diameter of the synthesized CNPs and CCNPs were found to be ~299 nm and ~355 nm, respectively. CNP and CCNP nanoparticles showed an intermediate polydisperisty index of 0.434 and 0.487, respectively (Figure 1a,b). However, the CCNPs were found to be spherical with sizes ranging from 130–341 nm as indicated by the TEM micrograph (Figure 1c). On comparing the functional groups present in the CCNPs with their bulk counterparts, chitosan showed characteristic peaks at 3418 and 3238 cm^−1^, which indicated the O-H stretching and N–H stretching vibration, the characteristic peak at 2908 cm^−1^ depicted the C–H stretch, 2342 cm^−1^ (C–N band stretching), 1610 cm^−1^ (amide II band), 1024 and 1051 cm^−1^ indicated that the CH_2_ group and C–O stretch from glucosamine residue was observed. Chrysin showed characteristic bands at 2625 cm^−1^, 2343 cm^−1^ indicating O–H stretching vibration and intramolecular H-bond (Figure 1d). The characteristic peaks of both chitosan (at 2342 cm^−1^, 1051 cm^−1^) and chrysin (at 2625 cm^−1^ and 2343 cm^−1^) were observed in the CCNPs. A slight band shift was also observed at 3347 and 3200 cm^−1^ to the lower wave-number that indicated the presence of hydrogen bonding between O-H group of chrysin and O-H or -NH_2_ group of chitosan [24]. The other peaks observed in the loaded nanoparticles were the P–O bending peak at 890 cm^−1^ and at 2625 cm^−1^, which was broader compared to pure chrysin, indicating an increase in hydrogen bond interactions [13].

### 3.2. Loading Efficiency and Release Kinetics of Chrysin-Loaded Chitosan NPs

**Figure 2:**
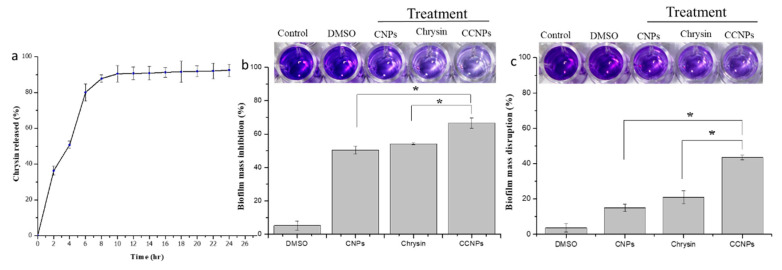
Drug release kinetics and Anti-biofilm assay: (**a**) In vitro release profile of chrysin from CCNPs at 37 °C and pH 7.4, the data are presented as mean ± SD; Graph showing biofilm (**b**) inhibition and (**c**) disruption on treatment with DMSO, CNPs, chrysin and CCNPs.

The amount of chrysin loaded onto the CCNPs was found to be 80.86 ± 0.30%. The in vitro drug release profile of chrysin from the CCNPs was determined in a release medium constituting PBS and DMSO, at 37 °C. The pH of the media was set at 7.4, as the ionic strength of the media plays a vital part in the stability and drug release. The cumulative chrysin release as a function of time is depicted in Figure 2a. The drug release kinetics of the loaded NPs initially showed a burst release which was followed by a steady and sustainable release from the 8th h. The first burst release was observed in the first two hours with 36.33 ± 1.58% of chrysin release. The second burst was observed after the sixth hour with 80.11% ± 0.84% drug release. The graph takes the form of a plateau starting from the 10th h to 24th h. It was also revealed that about a total of 90.5% ± 0.50% of the chrysin was released from the NPs within 10 h.

### 3.3. Minimum Inhibitory Concentration (MIC) and Sub-MIC of Chrysin-Loaded Chitosan NPs

The MIC value of the CCNPs was determined to be 1024 µg/mL for *S. aureus*. At 768 µg/mL, the NPs that did not exert any effect on the growth of the test bacteria. Hence, 768 µg/mL was selected as a sub-MIC concentration and used in all the subsequent anti-biofilm assays.

### 3.4. In Vitro Anti-Biofilm Activity of Chrysin-Loaded Chitosan NPs

#### 3.4.1. Crystal Violet Staining Assay for Biofilm Formation and Disruption

The CCNPs showed a reduction in biofilm formation as compared to its bulk counterparts. The biofilms were inhibited to 50.48 ± 2.42% and 54.1 ± 0.56 % on treatment with CNPs and chrysin, respectively (Figure 2b). However, the CCNPs inhibited the biofilm formation to 66.59 ± 3.09%. The treatment of the preformed biofilm with CCNPs also resulted in a reduction in the biofilm mass of 43.50 ± 1.29%, whereas a decrease in biofilm of 14.92 ± 2.17% and 20.94 ± 3.73% was observed in the presence of CNPs and chrysin, respectively (Figure 2c).

#### 3.4.2. Microscopic Examination of Biofilm

**Figure 3:**
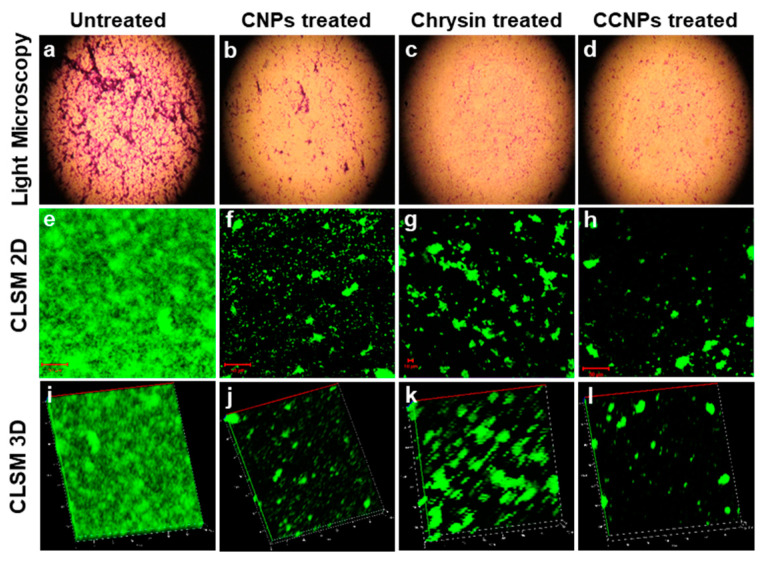
Microscopic examination of biofilm: Light microscopy images of *S. aureus* biofilm (**a**) untreated, and treated with (**b**) CNPs, (**c**) chrysin and (**d**) CCNPs, showing dispersion in biofilm formation. CLSM 2D images (**e**–**h**) and 3D images (**i**–**l**) showing bacterial biofilm untreated, and treated with CNPs, chrysin and CCNPs, respectively.

Light microscopy and CLSM were used to observe the change in the biofilm architecture of *S. aureus* in presence and absence of CCNPs. The influence of CCNPs on the thickness of biofilm, overall structure and biofilm density was evident in the micrographs. However, a dense biofilm was visible in the light microscope images of the untreated samples (Figure 3). A thick biofilm of 80 µm was observed in the control while the thickness was reduced to 20 µm on treatment with chrysin. However, a higher reduction in the thickness of the biofilm matrix to 16 µm was achieved in the presence of CCNPs (Figure 3).

#### 3.4.3. Exopolysaccharide (EPS) Quantification and Microbial Adhesion to Hydrocarbon (MATH) Assay

**Figure 4:**
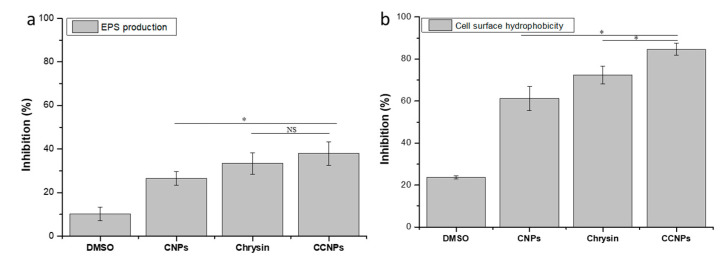
(**a**) Exopolysaccharide quantification and (**b**) Microbial adhesion to hydrocarbon assay: Effect of CNPs, chrysin, and CCNPs on the EPS production and cell surface hydrophobicity of *S. aureus*.

The CCNPs showed better reduction in the synthesis of EPS compared to its bulk counterparts. On treatment with CCNPs, a reduction in EPS production of 38.03 ± 5.41% was observed (Figure 4a). Chrysin and CNPs were also able to restrict the production of EPS by 33.37 ± 4.84% and 26.54 ± 3.20%, respectively. Cell surface hydrophobicity (CSH) is another important factor in biofilm formation as it aids in the adherence of the cell to the substratum. The CCNPs reduced the CSH in *S. aureus* by 84.66 ± 2.84% as compared to its bulk counter parts (Figure 4b). CNPs and Chrysin showed approximately 61.28 ± 5.78% and 72.46 ± 4.21% decreases in cell surface hydrophobicity, respectively. 

#### 3.4.4. Growth Curve Analysis 

**Figure 5:**
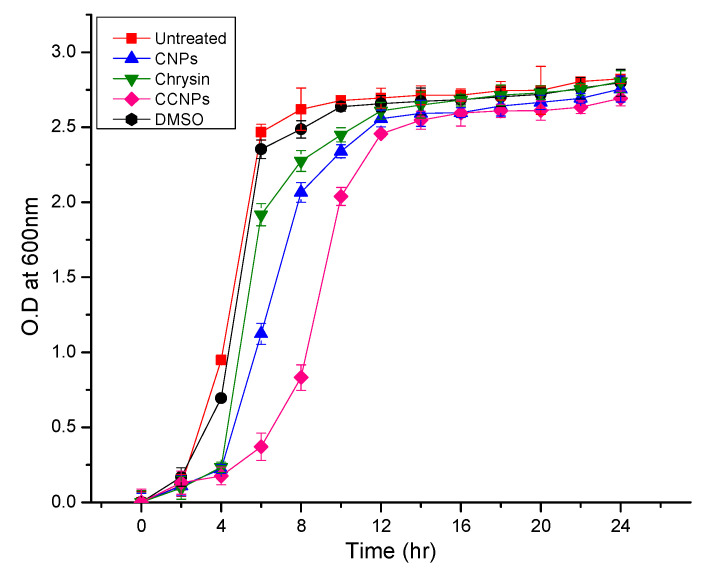
Growth curve analysis Growth curve of *S. aureus* incubated with chrysin, CNPs and CCNPs.

The growth pattern of the test organism when cultivated in the presence and absence of the CCNPs and CNPs is presented in Figure 5 Though the cells exhibited retardation in growth on exposure to a sub-MIC of the NPs and Chrysin, there was no significant decrease in the cell density. Hence, it can be inferred that the CCNPs arrested the biofilm development in *S. aureus*, indicating the potential application of CCNPs in the management of *S. aureus*-related infections.

## 4. Discussion

The formation of biofilm is one of the major obstacles in the modern antibacterial therapy. The biofilm-forming ability of bacteria provides the pathogen with advantages by blocking the entry of antimicrobial agents, thus causing hindrance in the clearance of these pathogens by the host immune system. Drug nanonization—i.e., a reduction in the particle size of drugs to nano-size—enhances the intracellular uptake of nanoparticles thus, providing a way to overcome the problems associated with insoluble drugs. Moreover, an increase in the surface area of poorly soluble drugs also leads to a more pronounced increase in the therapeutic index by maximizing the action with lesser dose [25]. In the present study, the anti-biofilm activity of CCNPs was demonstrated against biofilm forming bacterium, *S. aureus* MCC 2408. Chrysin was encapsulated to chitosan using the ionotropic gelation method. The NPs were formed due to the electrostatic interaction between amine group of chitosan and polyphosphate ions of TPP [19]. The hydrodynamic size influences various properties such as loading efficiency, drug release kinetics and the stability of the NPs. Though smaller nanoparticles due to high surface area show greater encapsulation efficiency, however, it also tends to aggregate easily on storage [19]. Particles of a low polydispersity index (PDI) are homogenous in nature and provide maximum stability. High polydispersity index (PDI) indicates the heterogeneity of the nanoparticles in the mixture. The synthesized spherical nanoparticles showed intermediate polydispersity that aids in the stability of the CCNPs. Ilk et al. [19] reported the synthesis of kaempferol loaded chitosan/TPP nanoparticles with an average particle size of 192.27 nm. The FTIR spectra of CCNPs indicated the presence of a similar functional group as that of its bulk counterpart indicating the successful encapsulation of chrysin with chitosan and the formation of chrysin-loaded chitosan NPs.

The biological efficacy of a drug and its potential use in drug delivery is directly influenced by the loading efficiency and controlled release. The CCNPs demonstrated a high encapsulation efficiency with a sustainable release. The CCNPs showed a higher loading efficiency compared to the previously synthesized nanocomposites such as the BSA-loaded chitosan-TPP nanoparticle with a loading efficacy of 60% [13] and PEG-chrysin conjugates with a loading efficacy of 55.6% [26]. The high encapsulation efficiency of CCNPs may be attributed to the presence of hydrogen bond between the -OH group of chrysin and the -NH2 group of chitosan that help in better entrapment of chrysin into the CNPs. From these release kinetics, it can be interpreted that chrysin was not covalently bonded to the nanoparticle and was thus easily released when dislodged in the medium. A similar result was observed in kaempferol-loaded chitosan nanoparticles, where more than 85% of the drug release was attained within 4 h, and no significant quantity of the drug was released thereafter [19].

Biofilm formation by *S. aureus* is associated with many nosocomial as well as chronic diseases associated with medical devices and surgical implants. It also leads to the emergence of the multi-drug resistant (MDR) strains viz. Methicillin-resistant *S. aureus* (MRSA) and Vancomycin-resistant *S. aureus* (VRSA) [3]. It was found that both CNPs and chrysin exhibited significant anti-biofilm activity relative to the untreated control. However, the anti-biofilm efficacy was comparatively enhanced when chrysin was encapsulated with chitosan. The ability to attach and establish biofilm on inert surfaces contributes to making *S. aureus* a major pathogen of chronic infections [10]. The data also suggested that the CCNPs showed better biofilm inhibition ability than disruption of preformed biofilm. Shi et al. [10] suggested that chitosan-coated iron oxide nanoparticles have the potential to effectively prevent bacterial colonization and control the biofilm formation by 53% in *S. aureus*. The anti-biofilm efficacy of the NPs was also validated by light and CLSM micrographs which showed a reduction in thickness and density of the biofilm matrix in presence of CCNPs.

The EPS matrix plays an indispensable role in the initial cell attachment, the formation of biofilm architecture and in providing mechanical stability of the biofilm. The EPS produced by the biofilm-forming bacteria prevents the access of antimicrobial agents and antibiotics to the bacterial cell [18]. The CCNPs caused a considerable decrease in the EPS production and cell surface hydrophobicity in *S. aureus* which resulted in the decrease in bacteria accumulation and attachment to the substratum. Hence, it can be inferred that CCNPs have a profound effect on the early stages in biofilm formation, specifically in the adherence and colonization as compared to its bulk counterparts, chrysin and chitosan. 

From the growth curve analysis, it can be interpreted that at the sub-MIC level, the CCNPs exerted less bactericidal effect and selective pressure against *S. aureus*. However, it had a profound effect on the *S. aureus* in the biofilm mode of growth. Chrysin and chitosan are found to be nontoxic in recommended concentrations. It was reported that the recommended daily concentration of this flavone is 0.5 to 3 g [27]. Likewise, chitosan nanoparticles are nontoxic at low concentrations and found to be toxic only at higher concentrations [28]. The nontoxic nature of these components may enable the application of CCNPs for biomedical applications. As these the outcome of the study suggested that the nanoformulation of chrysin exhibits enhanced synergistic anti-biofilm activity against *S. aureus* when compared to its bulk counterparts—chrysin and chitosan taken separately. Hence, CCNPs may be considered as a potential therapeutic agent for controlling biofilm formation in *S. aureus*. The nanocomposites may be further exploited towards the development of anti-biofilm coatings.

## 5. Conclusions

This study displayed an enhanced antibiofilm activity of chrysin against *S. aureus* when loaded on to chitosan-TPP nanoparticles, with a profound loading capacity. Chrysin-loaded chitosan nanoparticles were characterized to confirm the effective loading of the flavone on chitosan nanoparticles. Anti-biofilm activities of CCNPs were determined through biofilm inhibition, biofilm disruption, EPS reduction and hydrophobicity reduction assays. CCNPs synthesized could be used as a potential therapeutic agent for controlling biofilm formation in *S. aureus* in the future. The nanocomposites may be further exploited towards the development of anti-biofilm coatings.
