# Correction: Siddhardha et al. Chrysin-Loaded Chitosan Nanoparticles Potentiates Antibiofilm Activity against *Staphylococcus aureus*. *Pathogens* 2020, *9*, 115

**DOI:** 10.3390/pathogens13010029

**Published:** 2023-12-28

**Authors:** Busi Siddhardha, Uday Pandey, K. Kaviyarasu, Rajasekharreddy Pala, Asad Syed, Ali H. Bahkali, Abdallah M. Elgorban

**Affiliations:** 1 Department of Microbiology, School of Life Sciences, Pondicherry University, Puducherry 605014, India; uday.pandey@niser.ac.in; 2 Nanosciences African Network (NANOAFNET), Materials Research Group (MRG), iThemba LABS-National Research Foundation (NRF), Old Faure Road, P.O. Box 722, Somerset West 7129, South Africa; kasinathankaviyarasu@gmail.com; 3 Department of Biomedical & Pharmaceutical Sciences, Chapman University, School of Pharmacy, Irvine, CA 92618-1908, USA; rrpala@chapman.edu; 4 Department of Botany and Microbiology, College of Science, King Saud University, P.O. 2455, Riyadh 11451, Saudi Arabia; abahkali@ksu.edu.sa (A.H.B.); aelgorban@ksu.edu.sa (A.M.E.)

## Error in Figure

The authors wish to make the following corrections to the original publication [1]. The authors regret that an error was found in Figure 3 of the above article. We noticed mistake in the orientation of confocal laser scanning microscope (CLSM) images of *S. aureus* biofilm in Figure 3. The following figure shows corrected CLSM 2D images (Figure 3e–h) with their respective 3D images (Figure 3i–l). The correct version of Figure 3 appears below: 

We stress that these errors were purely due to human error and oversight; the corrections made do not affect or change the written portion of the figure legend, the interpretation of the results or the final conclusions of this manuscript. The authors would like to apologize for any inconvenience caused and state that the scientific conclusions are unaffected. This correction was approved by the Academic Editor. The original publication has also been updated.

## Figures and Tables

**Figure 3:**
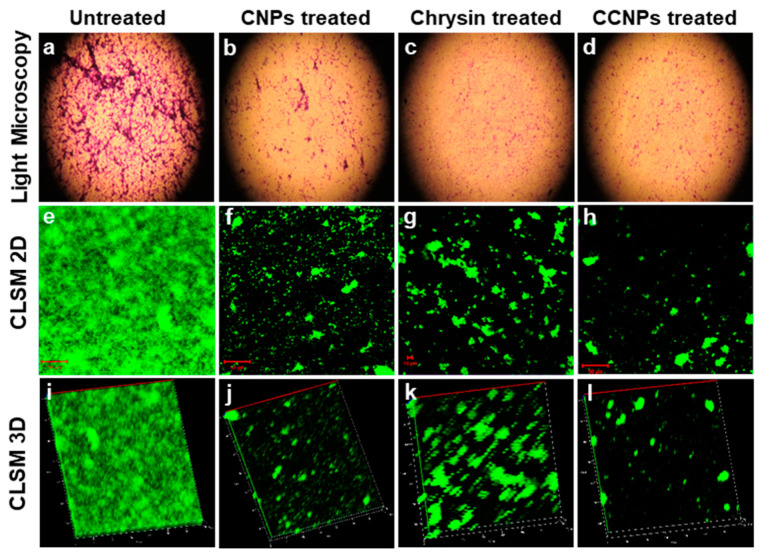
Microscopic examination of biofilm: Light microscopy images of *S. aureus* biofilm (**a**) untreated, and treated with (**b**) CNPs, (**c**) chrysin and (**d**) CCNPs, showing dispersion in biofilm formation. CLSM 2D images (**e**–**h**) and 3D images (**i**–**l**) showing bacterial biofilm untreated, and treated with CNPs, chrysin and CCNPs, respectively.
